# Índices Nutricionais e Fibrilação Atrial Pós-Operatória: Índice Triglicerídeos-Colesterol-Peso Corporal

**DOI:** 10.36660/abc.20250171

**Published:** 2025-05-06

**Authors:** Zulkif Tanriverdi

**Affiliations:** 1 Harran University Faculty of Medicine Department of Cardiology Sanliurfa Turquia Department of Cardiology, Faculty of Medicine, Harran University, Sanliurfa – Turquia

**Keywords:** Fibrilação Atrial, Avaliação Nutricional, Triglicerídeos

A fibrilação atrial pós-operatória (FAPO) é uma das complicações mais comuns após cirurgia cardíaca e a causa mais comum de fibrilação atrial (FA) secundária.^1^ Embora ocorra em taxas diferentes dependendo do tipo de operação cardíaca, foi relatado que ocorre em aproximadamente 20% dos pacientes após cirurgia de revascularização do miocárdio (CRM).^2^ Apesar dos avanços tecnológicos e de todas as estratégias preventivas desenvolvidas, ainda é relativamente comum.^1,3^ A FAPO aumenta a duração e o custo da hospitalização e está associada ao aumento da morbidade e mortalidade. Também pode ser um preditor do desenvolvimento de FA não cirúrgica nos anos após a cirurgia cardíaca.^2^ Portanto, é importante antecipar os pacientes que podem desenvolver FAPO após a cirurgia cardíaca.^1^

Até o momento, muitos biomarcadores séricos foram investigados para identificar pacientes em risco de FAPO.^4^ Recentemente, foi demonstrado que a desnutrição é prevalente em pacientes hospitalizados, e se descobriu que a desnutrição está associada a uma maior incidência de FA e resultados cardíacos adversos devido à desaceleração do processo de recuperação e ao aumento do risco de complicações.^5,6^ Muitas ferramentas diferentes de triagem nutricional, das mais simples às bastante complexas, estão disponíveis para avaliar a desnutrição na prática clínica diária.^7^ Alguns novos índices nutricionais foram desenvolvidos por parâmetros objetivamente mensuráveis, como triglicerídeos, colesterol total, peso corporal, albumina, linfócitos e variáveis laboratoriais adicionais para identificar o estado nutricional. Embora vários sistemas e pontuações tenham sido avaliados em estudos clínicos, 4 índices nutricionais objetivos têm sido comumente propostos para a avaliação da desnutrição, como segue: índice de risco nutricional geriátrico (GNRI), índice de nutrição prognóstica (PNI), índice de triglicerídeos-colesterol-peso corporal (ITCC) e o escore de controle do estado nutricional (CONUT).^8^

O ITCC é o índice nutricional mais recentemente desenvolvido entre esses índices. Foi introduzido pela primeira vez por Doi et al. em 2018.^9^ É um parâmetro fácil de usar, simples, rápido, mensurável e barato para avaliar a desnutrição na prática clínica. Estudos examinaram a significância clínica do ITCC e mostraram que ele não é apenas um índice que mostra a nutrição, mas também pode prever o prognóstico em diferentes doenças.^6,9-12^ Além disso, em um estudo recente, Doi et al. descobriram que o ITCC também poderia ser usado como um marcador prognóstico valioso em pacientes submetidos à implantação de válvula aórtica transcateter.^13^ No entanto, são limitados os dados disponíveis sobre a relação entre o ITCC e a FAPO em pacientes submetidos à cirurgia de CRM.

Nesta edição dos Arquivos Brasileiros de Cardiologia, Koyuncu et al.^14^ focaram no impacto do estado nutricional nos resultados cirúrgicos e investigaram o papel do ITCC como preditor de FAPO. Os autores examinaram 321 pacientes submetidos a CRM por um longo período de 14 anos e relataram que a FAPO foi desenvolvida em 62 (19,3%) pacientes após CRM, o que foi consistente com a literatura.^1^ Além disso, foi descoberto que a idade, a frequência de hipertensão, proteína C-reativa (PCR), leucócitos e ITCC foram significativamente maiores em pacientes que desenvolveram FAPO. Na análise de regressão logística multivariada, maior idade e menor ITCC foram detectados como preditores independentes de FAPO. O possível mecanismo subjacente foi descrito como o mau estado nutricional que pode exacerbar o processo inflamatório que desempenha um papel importante no desenvolvimento de FA.^14^

Existem estudos limitados sobre o efeito da nutrição avaliada por parâmetros objetivamente mensuráveis no desenvolvimento de FAPO após cirurgia cardíaca. Nesses estudos, se descobriu que valores mais baixos de PNI e GNRI estavam significativamente associados à maior incidência de FAPO em pacientes submetidos à cirurgia cardíaca.^7,15,16^ Além disso, um escore mais baixo de GNRI foi associado ao aumento de recorrências de arritmia em pacientes submetidos à ablação por cateter para FA.^17^ Os resultados desses estudos demonstraram que a desnutrição pré-operatória está intimamente relacionada ao desenvolvimento de FA após operações cardíacas. No entanto, nenhum estudo anterior investigou o ITCC para a predição de FAPO em pacientes submetidos à CRM. O estudo de Koyuncu et al.^14^ parece ser o primeiro estudo sobre a relação entre ITCC e o desenvolvimento de FAPO e, portanto, fornece uma contribuição significativa para a literatura.

Consequentemente, a desnutrição é uma condição clínica importante que pode ser facilmente negligenciada quando não avaliada por parâmetros objetivos e mensuráveis, mas deve ser mantida em mente em pacientes hospitalizados, especialmente aqueles submetidos a cirurgia cardíaca. Dada a prevalência da desnutrição e seu impacto na duração da internação hospitalar, custos e resultados adversos, a detecção e o tratamento precoces melhorarão o prognóstico. Em pacientes com pontuações baixas e submetidos a cirurgia cardíaca, se deve considerar um suporte nutricional mais intensivo e o uso de terapias preventivas no período pré-operatório, e esses pacientes devem ser acompanhados mais de perto para o desenvolvimento de FAPO.
