# Supplementary material for: Índices Nutricionais e Fibrilação Atrial Pós-Operatória: Índice Triglicerídeos-Colesterol-Peso Corporal
Source: Arq Bras Cardiol. 2025 May 6;122(4):e20250171. [Article in Portuguese] doi: 10.36660/abc.20250171 (PMC12097714; doi:10.36660/abc.20250171)
Supplement: Translation

# Translation: Nutritional Indices and Postoperative Atrial Fibrillation: The Triglyceride-Cholesterol-Body Weight Index

**DOI:** 10.36660/abc.20250171i

**Authors:** Zulkif Tanriverdi

**Affiliations:** 1 Department of Cardiology Faculty of Medicine Harran University Sanliurfa Türkiye Department of Cardiology, Faculty of Medicine, Harran University, Sanliurfa – Türkiye

**Keywords:** Atrial Fibrillation, Nutrition Assessment, Triglycerides

Postoperative atrial fibrillation (POAF) is one of the most common complications after cardiac surgery and the most common cause of secondary atrial fibrillation (AF).^1^ Although it occurs at different rates depending on the type of cardiac operation, it has been reported to occur in approximately 20% of patients after coronary artery bypass grafting (CABG).^2^ Despite technological advances and all the preventive strategies developed, it is still relatively common.^1,3^ POAF increases the length and cost of hospitalization and is associated with increased morbidity and mortality. It may also be a predictor of non-surgical AF development in the years after the cardiac surgery.^2^ Therefore, it is important to anticipate patients who may develop POAF after the cardiac surgery.^1^

To date, many serum biomarkers have been investigated to identify patients at risk for POAF.^4^ Recently, it has been demonstrated that malnutrition is prevalent in hospitalized patients, and malnutrition has been found to be associated with a higher incidence of AF and adverse cardiac outcomes due to slowing down the recovery process and increasing the risk of complications.^5,6^ Many different nutritional screening tools, from very simple to quite complex, are available to assess malnutrition in daily clinical practice.^7^ Some novel nutritional indices have been developed by objectively measurable parameters such as triglyceride, total cholesterol, body weight, albumin, lymphocyte, and additional laboratory variables to identify nutritional status. Although various systems and scores have been assessed in clinical studies, 4 objective nutritional indices have commonly been proposed for the evaluation of malnutrition as follows: geriatric nutritional risk index (GNRI), prognostic nutrition index (PNI), triglyceride-cholesterol-body weight Index (TCBI) and controlling nutritional status score.^8^

TCBI is the most recently developed nutritional index among these indexes. It was first introduced by Doi et al. in 2018.^9^ It is an easy-to-use, simple, rapid, measurable, and cheap parameter to assess malnutrition in clinical practice. Studies have examined the clinical significance of the TCBI index and shown that it is not only an index that shows nutrition but also can predict prognosis in different diseases.^6,9-12^ Moreover, in a recent study, Doi and colleagues found that TCBI could also be used as a valuable prognostic marker in patients undergoing transcatheter aortic valve implantation.^13^ However, limited data are available regarding the relationship between the TCBI and POAF in patients undergoing CABG surgery.

In this issue of the *Arquivos Brasileiros de Cardiologia*, Koyuncu et al.^14^ focused on the impact of nutritional status on surgical outcomes and investigated the role of the TCBI as a predictor of POAF. The authors examined 321 patients who underwent CABG over a long period of 14 years and reported that POAF was developed in 62 (19.3%) patients after CABG, which was consistent with the literature.^1^ In addition, it was found that age, the frequency of hypertension, C-reactive protein (CRP), leukocytes, and TCBI were significantly higher in patients who developed POAF. In multivariate logistic regression analysis, higher age and lower TCBI were detected as independent predictors of POAF. The possible underlying mechanism has been described as poor nutritional status may exacerbate the inflammatory process that plays an important role in the development of AF.^14^

There are limited studies regarding the effect of nutrition evaluated by objectively measurable parameters on the development of POAF after cardiac surgery. In these studies, it was found that lower PNI and GNRI values were significantly associated with the higher incidence of POAF in patients undergoing cardiac surgery.^7,15,16^ In addition, a lower GNRI score was found to be associated with increased arrhythmia recurrences in patients who underwent catheter ablation for AF.^17^ Results of these studies have demonstrated that preoperative malnutrition is closely related to the development of AF after cardiac operations. However, no previous study investigated the TCBI for the POAF prediction in patients undergoing CABG. The study of Koyuncu et al.^14^ appears to be the first study regarding the relationship between TCBI and the development of POAF and, therefore, provides a significant contribution to the literature.

Consequently, malnutrition is an important clinical condition that can be easily overlooked when not assessed by objective, measurable parameters but should be kept in mind in hospitalized patients, especially those undergoing cardiac surgery. Given the prevalence of malnutrition and its impact on length of hospital stay, costs, and adverse outcomes, early detection and treatment will improve prognosis. In patients with low scores and undergoing cardiac surgery, more intensive nutritional support and the use of preventive therapies in the preoperative period should be considered, and these patients should be followed more closely for the development of POAF.
